# Endocarditis prophylaxis in children and adolescents: indications in consideration of the dental treatment setting

**DOI:** 10.1007/s00431-025-06410-3

**Published:** 2025-08-21

**Authors:** Maria Hofmann, Thomas Zajonz, Matthias Müller, Norbert Krämer, Nelly Schulz-Weidner

**Affiliations:** 1 Department of Pediatric Dentistry, Justus Liebig University, Schlangenzahl 14, 35392 Giessen, Germany; 2 Department of Anesthesiology, Operative Intensive Care Medicine, and Pain Therapy, Pediatric Heart Centre, University Hospital of Giessen and Marburg, Campus Giessen, Rudolf-Buchheim-Str. 7, 35392 Giessen, Germany

**Keywords:** Infective endocarditis, Heart disease, Dental treatment, Recommendation, Endocarditis prophylaxis

## Abstract

This study aimed to evaluate the administration of endocarditis prophylaxis (EP) in children and adolescents with cardiac findings requiring invasive dental therapy according to the newest European Society of Cardiology (ESC) guidelines from 2023. The research question focused on comparing EP protocols between chairside treatment (CS) and general anesthesia (GA) settings, given clinical protocol differences observed between these modalities. This retrospective analysis included 201 patients with cardiac findings who attended the Department of Pediatric Dentistry at Giessen University Hospital between 2018 and 2023 and underwent dental interventions with bacteremia risk. Patient data included cardiac findings, EP administration protocols, antibiotic agents used, treatment settings, and patient outcomes defined as infective endocarditis (IE) occurrence within three months post-intervention. EP indications were evaluated according to ESC guidelines. Fisher’s exact test was used to statistically test differences (GA vs. CS) regarding the outcome and the type of EP medication (*α* = 0.05). Of all patients, 58.7% received treatment under GA and 41.3% chairside. According to ESC guidelines, EP indication existed in 36.4% of GA patients and 22.9% of CS patients. However, EP was administered in 84.7% of GA patients and 59.0% of CS patients. A significant correlation was found between the variables “treatment setting” and “antibiotic preparation EP” (*p* < 0.001). No cases of IE occurred within 3 months, regardless of EP administration or indication status.

*Conclusion*: Besides the limitations of our retrospective study, frequent EP over-administration beyond guideline recommendations was detected, and no IE cases occurred. This aspect could question whether EP indications should be more restrictively defined, particularly in children and adolescents with healthy dental status.

**Table:** 

**What is Known:** • *The indication for antibiotic endocarditis prophylaxis (EP) during dental treatment is based on cardiac and dental risk factors.* • *In 2023, the European Society of Cardiology (ESC) published new guidelines for the prevention of infective endocarditis (IE) [1].*
**What is New:** • This study highlights the difficulties in implementing guideline-adhering endocarditis prophylaxis and emphasizes the need for strict and comprehensible EP indication in children and adolescents.

**What is Known:**

• *The indication for antibiotic endocarditis prophylaxis (EP) during dental treatment is based on cardiac and dental risk factors.*

• *In 2023, the European Society of Cardiology (ESC) published new guidelines for the prevention of infective endocarditis (IE) [1].*

**What is New:**

• This study highlights the difficulties in implementing guideline-adhering endocarditis prophylaxis and emphasizes the need for strict and comprehensible EP indication in children and adolescents.

## Introduction

Infective endocarditis (IE) is a rare condition that is furthermore associated with a high mortality rate [1] and represents one of the most severe complications associated with dental procedures, caused by iatrogenic bacteraemia mediated by oral bacteria [2]. To prevent this, patient groups at high risk for developing IE should receive antibiotic prophylaxis prior to certain dental procedures [3]. Guidelines regarding endocarditis prophylaxis (EP) and its indications differed and differ between societies, countries, and expert opinions [4]. In 2007, the latest edition of the American Heart Association (AHA) guidelines for EP was published [3]. The German position paper on “Prophylaxis of infective endocarditis,” also from 2007, mainly follows these guidelines [5]. The most recent SK-2 guideline “Infective endocarditis and endocarditis prophylaxis” by the German Society for Pediatric Cardiology and Congenital Heart Defects from 2022 [6] refers to the 2007 position paper regarding recommendations for antibiotic endocarditis prophylaxis. In 2023, new guidelines for the management of IE were published by the European Society of Cardiology (ESC) [7]. However, these are not consistent with those of the AHA. According to ESC guidelines, patients with transcatheter valve prostheses are explicitly included in the indication group; there is no longer a general indication for EP in heart transplant patients with valvular dysfunction, and simply corrected heart defects without the use of prosthetic material and without existing shunts are excluded from the indication group. The range of procedures was also restricted even more strictly to invasive dental procedures, and clindamycin was excluded as an alternative medication. However, the ESC recommends prophylaxis rather than the AHA for some complex congenital heart defects after surgery (such as right ventricle to pulmonary artery conduits). The various recommendations make standardized implementation of EP difficult.

EP indications are being increasingly restricted so that only high-risk groups for the development of IE should receive EP. Patients with a pre-existing heart disease, for example, specific congenital heart defects, are described as high-risk patients. Procedures with a risk of IE today include almost only dental treatments and, among these, those with a risk of bacteraemia [3, 7].

The aim of the study was therefore to evaluate the administration of EP in children and adolescents with cardiac findings and necessary invasive dental therapy according to the newest guidelines of the ESC (2023) [7]. Since clinical experience often reveals protocol differences depending on dental treatment setting (chairside treatment [CS] or treatment under general anesthesia [GA]), data collection and analysis were performed separately for CS and GA.

## Materials and methods

This retrospective analysis included all patients with congenital heart defects who attended the Department of Pediatric Dentistry at Giessen University Hospital between 2018 and 2023 and who had undergone at least one dental intervention with a risk of bacteraemia. The study was approved by the local ethics committee of Medicine of the Justus Liebig University, Giessen (AZ 24/24). The patient data collected included basic patient parameters, cardiac, dental, and treatment-specific target parameters, as well as patient outcome defined as the occurrence of an IE within three months after the dental intervention. The patient baseline parameters included the patient’s age at the time of dental intervention and the patient’s gender. The type of the individual cardiac finding, EP indication according to the applied guidelines (ESC vs. AHA), documentation of the administration of EP, prophylactic agent used, and the pediatric department administering the prophylaxis, as well as dosage of EP, were documented as cardiac target parameters. The patient’s cardiac findings were extracted from the last available, preferably cardiological, doctor’s letter or the written patient history prior to the dental intervention in the patient data management systems (PDMS; MEONA [Mesalvo Freiburg GmbH, Freiburg, Germany], EVIDENT [EVIDENT GmbH, Bad Kreuznach, Germany], and the treatment documentation software “Multizentrische Dokumentation” [MZD] of the dental clinic). The type of cardiac finding was categorized according to Warnes et al. (2001) [8] into four categories: “simple cardiac defect,” “moderate cardiac defect,” “complex cardiac defect,” or “other” (= cardiac findings that are not congenital, macroscopic anatomical abnormalities of the heart). Combinations of heart defects that would have been categorized as “mild heart defects” and corrected heart defects that exceeded the extent of the “mild heart defects” category and were not clearly listed in the Warnes et al. categories were categorized as “moderate heart defect.” EP indication was checked by an experienced pediatric cardiac anesthesiologist of the Pediatric Heart Centre of the Giessen University Hospital. Parameters such as dental treatment needs (“odontogenic infection,” “dmft/DMFT value” [= number of primary and permanent decayed, missing, and filled teeth due to caries] as information on the caries history and newly carious lesions; identified by pediatric dentists), the type of dental intervention, the type of patient care, and treatment setting were recorded as secondary target parameters. It was also documented whether the respective patient was known to have a penicillin allergy and if EP indication was recorded in the doctor’s letter. Moreover, it was documented whether IE occurred within 3 months after dental intervention. All data collected were saved in electronic tabular form (Microsoft Excel 2016, Microsoft Corporation, Redmond, WA, USA) while the dental clinic served as the data warehouse. The statistical analysis was carried out using the software programme “IBM® SPSS® Statistics 29.0.2.0” (IBM, Armonk, NY, USA). Fisher’s exact test was used to statistically test differences between treatment groups (GA/CS) regarding the outcome and the type of EP medication. A significance level (*α*) of 5% was defined for the entire inductive statistical analysis.

## Results

**Fig. 1:**
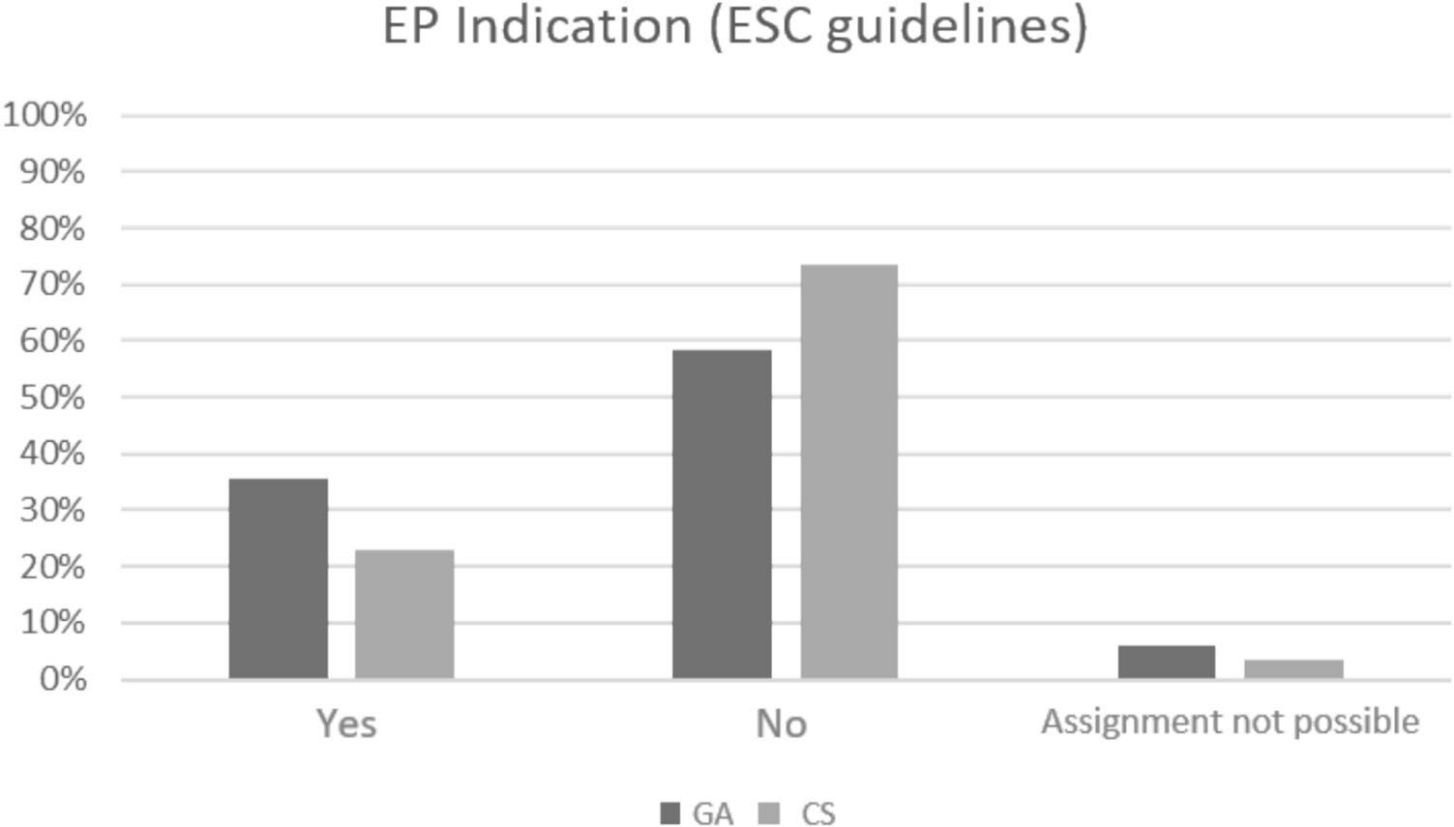
Relative frequencies of the assignment of endocarditis prophylaxis (EP) indication according to the European Society of Cardiology (ESC) guidelines for the study population based on the cardiac findings present in each case, divided according to the treatment setting. GA (*n* = 118), CS (*n* = 83). Overall, there was no EP indication in more than half of the cases in both groups. There was a more frequent indication in GA patients than in CS patients

A total of *n* = 201 patients met the inclusion criteria (January 2018–December 2023). Of these patients, 58.7% received dental treatment under GA, whereas 41.3% were treated chairside. Table 1 provides detailed information on the age and gender distribution for the respective treatment setting and patient care. A total of 23.9% of the patients presented simple, 41.3% moderate, and 27.9% complex congenital heart defects. The category “other” was assigned to 7%. According to the ESC guidelines, there was EP indication in 36.4% for GA and in 22.9% in CS. There was no EP indication in 73.5% of CS patients and in 57.6% in the GA group. A total of ten patients (5.9% GA and 3.6% CS) lacked information in the PDMS regarding the cardiac findings to clearly assign the respective case to an indication. A visual representation of the relative frequencies of the assignment of EP indication can be found in Fig. 1.

**Table 1:** Cross-tabulation: age and gender distribution as well as “type of patient care” (absolute [*N*] and relative [%] frequencies) and dental parameters (“odontogenic infection,” “DMFT/dmft value,” “fresh caries lesions”) of the study population; subdivided according to “treatment setting.” *SD*, standard deviation; *GA*, general anesthesia; *CS*, Chairside

			Treatment setting		Total
			GA	CS	
Gender	Male	*N*	60	46	106
		(%)	(50.8)	(55.4)	(52.7)
	Female	*N*	58	37	95
		(%)	(49.2)	(44.6)	(47.3)
Age (years)	Mean		7.8	10.3	8.8
	SD		3.9	4.4	4.3
	Minimum		2.1	2.0	2.0
	Maximum		18.3	18.6	18.6
Type of patient care	Inpatient	*N*	115	1	116
		(%)	(97.5)	(1.2)	(57.7)
	Outpatient	*N*	3	82	85
		(%)	(2.5)	(98.8)	(42.3)
Odontogenic infection	Fistula	*N*	11	5	16
		(%)	(9.3)	(6.0)	(8.0)
	Abscess	*N*	4	4	8
		(%)	(3.4)	(4.8)	(4.0)
DMFT/dmft value	Mean		8.5	5.2	7.2
	SD		5.0	5.1	5.3
	Minimum		0	0	0
	Maximum		28	25	28
newly developed carious lesions	Mean		8.3	3.0	6.1
	SD		5.0	3.8	5.2
	Minimum		0	0	0
	Maximum		28	17	28

In the GA group, 36.4% who had documented EP indication was based on the presence of heart valve prostheses or prosthetic material in 15.3% of cases, a history of endocarditis in 2.5% of cases, and a non-corrected cyanotic heart defect in 17.8% of cases. In contrast, the indication in the CS group, 22.9%, was based on the presence of heart valve prostheses or prosthetic material in 9.3% of patients. Furthermore, 1.2% of the patients had a history of endocarditis, and 8.4% of patients suffered from an uncorrected cyanotic congenital heart defect. Irrespective of the treatment setting, instructions regarding the administration of EP were documented in the (cardiological) doctor’s letter in only 33.8% of cases. In the majority (63.2%), no information was given regarding the doctor’s letter. Moreover, 3.0% of the patients did not have a traceable letter. The indications and the associated justifications according to the ESC guidelines corresponded almost completely to the AHA guidelines. Only in one case, belonging to the GA group, was an isolated recommendation according to the ESC guidelines detected.

**Fig. 2:**
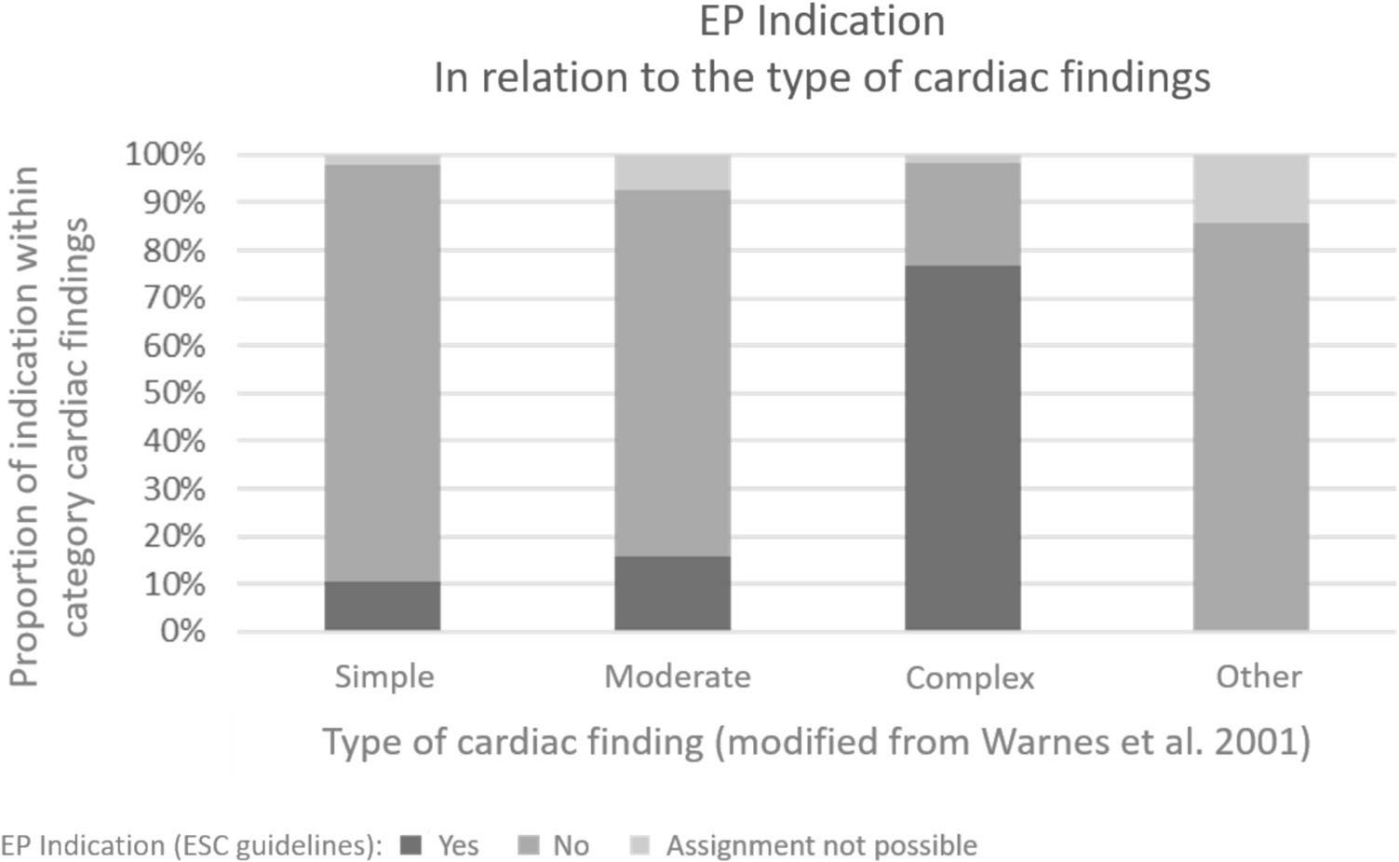
Relative frequencies of the variables for endocarditis prophylaxis (EP) indication according to European Society of Cardiology (ESC) guidelines (yes/no/no assignment possible) within the category “type of cardiac findings” (modified from Warnes et al. 2001 [8]). EP indication: yes (*n* = 62), no (*n* = 129),assignment not possible (*n* = 10).The EP indication increased with the complexity of the cardiac findings; in complex cardiac findings, there was most often an EP indication

In patients with simple cardiac findings, there was an EP indication according to ESC guidelines in 10.4%, in patients with moderate or complex cardiac findings in 15.7 and 76.8% of cases, respectively. There was no EP indication in any of the patient cases with cardiac findings in the “other” category. Figure 2 provides a graphical representation of the indication for the administration of an EP in relation to the type of cardiac findings.

In the GA group, EP was administered in 84.7%. In contrast, only 59.0% of the patients of the CS group received EP (see Table 2). In the GA group, all patients in whom EP was indicated according to ESC guidelines obtained a corresponding antibiotic shield. But also, 77.9% of patients without indication also received an EP. CS patients with EP indication received EP before the intervention in 78.9%. Patients from the CS group without indication were given EP in 52.5%, while 6.5% of all patients (all CS group) with EP indication did not receive any EP for the dental intervention. Overall, patients for whom no clear indication could be established due to a lack of information in the findings received an EP in 60.0%.

**Table 2:** Cross-tabulation: administration of antibiotic endocarditis prophylaxis (EP; absolute [*N*] and relative [%] frequency), split by treatment setting and indication according to ESC guidelines. *GA*, general anesthesia; *CS*, chairside

Treatment setting				EP administered?		Total
				Yes	No	
GA	Indication according to ESC guidelines	Yes	*N*	43	0	43
			(%)	(100.0)	(0.0)	(100.0)
		No	*N*	53	15	68
			(%)	(77.9)	(22.1)	(100.0)
		Assignment not possible	*N*	4	3	7
			(%)	(57.1)	(42.9)	(100.0)
	Total		*N*	100	18	118
			(%)	(84.7)	(15.3)	(100.0)
CS	Indication according to ESC guidelines	Yes	*N*	15	4	19
			(%)	(78.9)	(21.1)	(100.0)
		No	*N*	32	29	61
			(%)	(52.5)	(47.5)	(100.0)
		Assignment not possible	*N*	2	1	3
			(%)	(66.7)	(33.3)	(100.0)
	Total		*N*	49	34	83
			(%)	(59.0)	(41.0)	(100.0)
Total (GA and CS)	Indication according to ESC guidelines	Yes	*N*	58	4	62
			(%)	(93.5)	(6.5)	(100.0)
		No	*N*	85	44	129
			(%)	(65.9)	(34.1)	(100.0)
		Assignment not possible	*N*	6	4	10
			(%)	(60.0)	(40.0)	(100.0)
	Total		*N*	149	52	201
			(%)	(74.1)	(25.9)	(100.0)

With regard to dental treatment need parameters (DMFT/dmft value and newly developed carious lesions; see Table 1), 15 patients in the GA group and 9 patients in the CS group were affected by an odontogenic infection in the form of a fistula or an abscess. The average DMFT/dmft value was 8.5 (± 5.0) in the GA group and 7.2 (± 5.3) in the CS group. GA patients showed an average of 8.3 (± 5.0) and CS patients an average of 6.1 (± 5.2) newly developed carious lesions.

**Fig. 3:**
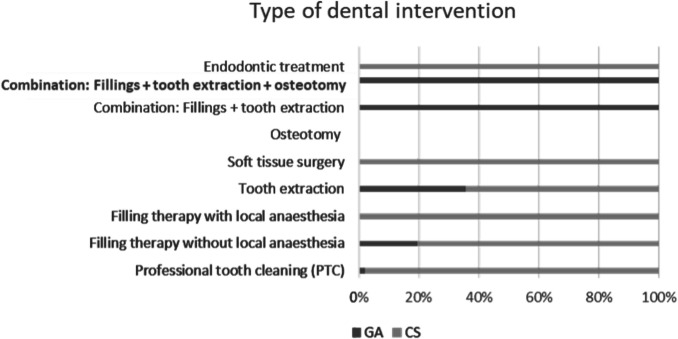
Type of dental intervention (relative frequency in %), split by treatment setting. GA (*n* = 118), CS (*n* = 83).Surgical and conservative combination therapies were only performed in the GA group. Professional tooth cleaning, isolated soft tissue surgery, and isolated filling therapies with local anesthesia were performed exclusively in the CS group

With the exception of a single case, professional dental cleanings were performed exclusively chairside. Professional dental cleanings accounted for the majority of chairside interventions (39.8%), followed by filling therapies without additional local anesthesia (31.3%) and tooth extractions (22.9%). For the group of patients treated under GA, combination therapy of fillings and tooth extractions was the most common type of intervention, accounting for 75.4% of all treatments, followed by tooth extractions alone (12.7%) and filling therapies without local anesthesia (7.6%). Combination therapy consisting of fillings and tooth extractions, with or without osteotomies, was performed exclusively in GA, while endodontic treatments were performed exclusively chairside. A visual representation of the distribution of dental interventions, allocated by treatment setting, can be found in Fig. 3.

In 98.0% of cases in which EP was administered, the antibiotic agent was documented. Of these, 96.6% received an aminopenicillin or a cephalosporin. According to the records, one patient (0.8%) in the GA group and two patients (2.4%) in the CS group were known to be allergic to penicillin. Patients with penicillin allergy received no EP in one case (reason unknown), amoxicillin and sulbactam in another case, and clindamycin in a further case.

**Fig. 4:**
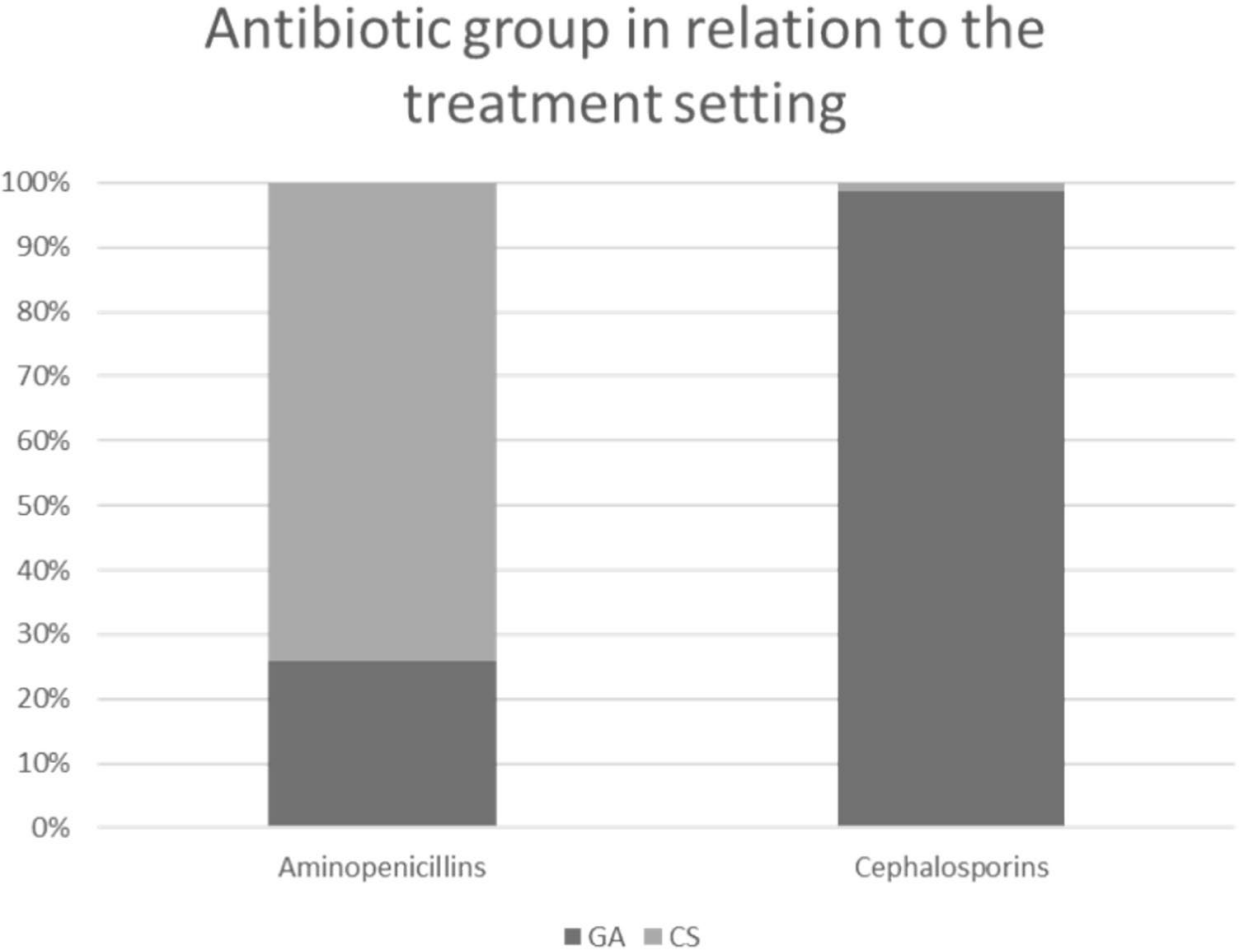
Antibiotic groups aminopenicillins and cephalosporins in relation to the treatment setting (GA/CS). GA (*n* = 118), CS (*n* = 83). The GA group received almost exclusively cephalosporins, while the CS group mainly received aminopenicillins

A significant correlation was found between the variables “treatment setting” and “antibiotic preparation EP” for the antibiotic groups aminopenicillins and cephalosporins (Fisher’s exact test; *p* < 0.001). Figure 4 provides a graphical representation of the distribution of the two antibiotic groups within the study subgroups (GA, CS).

For 83.9% of cases in the GA and 57.8% of cases in the CS group, the medical specialty of the doctor administering the EP and the chosen dosage form was documented in the patient file. Patients treated in GA received the EP in 59.6% by a pediatrician, in 40.4% by an anesthetist, and never by a dentist. Patients from the CS group received the EP in 95.8% by a dentist, in 4.2% by a pediatrician, and never by an anesthetist. In 97.9% of CS patients, EP was administered orally. EP was administered intravenously in 99.0% of those who received treatment under GA. Anesthetists administered the EP intravenously in 100.0% and pediatricians in 98.3% of cases. Dentists administered the EP orally.

There was no case of IE occurrence within three months after dental intervention. In 25.4% (GA group = 28.8%, CS group = 20.5%), no recall after dental treatment could be carried out.

## Discussion

In the present study, 201 patients of a tertiary care pediatric heart centre were retrospectively analysed with regard to EP protocols and the occurrence of IE in the context of dental interventions in a CS and GA setting. Moderate cardiac findings predominated (41.3%), followed by complex (27.9%) and mild (23.9%) conditions. This distribution contradicts German population patterns, where Lindinger et al. (2010) found mild defects predominating [9]. This discrepancy may reflect referral patterns, as patients with mild conditions often receive ambulatory care in private practices, while those with severe conditions present to clinics due to infrastructure requirements and superior complication management capabilities.

In more than half (57.0%) of all cases, no EP indication existed according to ESC guidelines. However, actual antibiotic prophylaxis administration in both study groups extended far beyond the high-risk patient population. EP was generally administered more frequently when patients were treated under GA. Despite the fact that the guidelines state that the decision to administer EP is ultimately at the individual discretion of the responsible practitioner, EP administration was associated with increasing complexity of the patient’s heart defect and GA. This may indicate that in-hospital recommendations or workflows are considered more relevant than evidence-based guidelines. This observation was not surprising, as various studies in the medical-antimicrobial-antibiotic setting have demonstrated [10–12].

The excessive administration of antibiotics must be viewed critically, particularly in view of the development of bacterial resistance [13]. Bacterial resistance constitutes a global challenge associated with high morbidity and mortality [14, 15]. Further investigation, particularly at microbial levels, is necessary to restrict EP to an even more limited intervention spectrum, preventing resistance development. Moreover, limited research exists regarding resistance development from repeated EP administration in children with cardiac conditions.

Establishing continuous interdisciplinary exchange between dentistry and medicine (cardiology, pediatrics, anesthesia) is essential for appropriate EP decision-making. Clinical decision support systems and computerized clinical decision support represent promising options for optimizing EP decision-making [11, 12].

Fisher’s exact test demonstrated a statistically significant systematic association between study groups (GA, CS) and antibiotic classes (aminopenicillins, cephalosporins) (*p* < 0.001). Cefuroxime predominated in GA patients (76.8%). Potential explanations include higher cephalosporin bioavailability with intravenous versus oral administration [16], though this does not explain why intravenous aminopenicillins were not equally utilized. Cephalosporins are recommended for both penicillin-allergic and non-allergic patients according to guidelines, potentially explaining their preference given the penicillin allergy prevalence of 5–20% [17]. Patients with penicillin allergy all received different prophylactic agents. One patient received guideline-recommended clindamycin. One case received penicillin despite documented allergy. The GA group received EP predominantly parenterally, prescribed by medical physicians, while the chairside group received primarily oral EP prescribed by dental practitioners. Since no IE was documented in any of the cases, the form of administration of the antibiotic agent as well as the antibiotic class did not indicate any influence on the occurrence of IE. Parenteral administration can primarily be attributed to the fact that GA patients received intravenous access preoperatively or as part of the anesthesiological induction to enable safe anesthesia. Aspects of cooperation and compliance and the possibility of subtotal enteral intake (negative taste experience, regurgitation, partial emesis) can thus be avoided.

Although the patients often showed high caries experience with many untreated newly developed carious lesions or even odontogenic fistulas and abscesses, IE did not occur as a result of dental therapy in any of the cases. The severity of the dental findings cannot be regarded as a predictor of IE. This is of high importance due to the fact that children with congenital heart disease demonstrate increased caries prevalence compared to healthy children [18]. These patients show higher DMFT/dmft values regardless of age group compared to healthy peers [19]. Larger treatment needs combined with younger age and reduced compliance, after periods of prolonged hospitalization due to corrective cardiac interventions, often indicate GA rehabilitation, potentially explaining higher GA case numbers. GA typically involves more invasive therapies than chairside treatment.

Study groups also differed regarding dental intervention types. Professional dental cleaning predominated in chairside treatment (39.8%), representing a procedure better tolerated by less cooperative patients than invasive treatments requiring local anesthesia. Positively, professional cleaning constitutes primary IE prophylaxis. Endodontic procedures occurred exclusively in chairside patients, reflecting their status as compromise treatments with uncertain prognosis. S2k guidelines recommend extraction over endodontic treatment for necrotic primary teeth to avoid postoperative infectious complications [20]. Three-quarters (75.4%) of GA patients received combination therapy (restorations and extractions), appearing logical for comprehensive treatment completion while avoiding additional GA in this vulnerable population. Neither invasive dental procedures, such as tooth extractions, nor professional dental cleanings resulted in IE.

A limitation of the retrospective design is that our study is not a valid method for causality research. However, patients cannot be randomized to GA or CS treatment, as patient-specific factors including age, compliance, and intervention complexity determine treatment setting [21]. On the other hand, IE represents a rare condition: every year, three to ten out of every 100,000 people are affected by IE [22]. The rarity of IE occurrence is confirmed by current study results, at least regarding dental interventions in children and adolescents. Given the rarity of IE, these results must be interpreted with caution. The small sample size necessitates examination of additional patients to draw more definitive conclusions. Future research could include retrospective analysis of confirmed endocarditis cases, specifically evaluating the prophylactic measures implemented, to increase the overall case volume and strengthen the evidence base.

## Data Availability

The data that support the findings of this study are available from the corresponding author upon reasonable request.

## Abbreviations

AHA: American Heart Association
CS: Chairside
EP: Endocarditis prophylaxis
ESC: European Society of Cardiology
GA: General anesthesia
IE: Infective endocarditis
PDMS: Patient data management system
SD: Standard deviation
